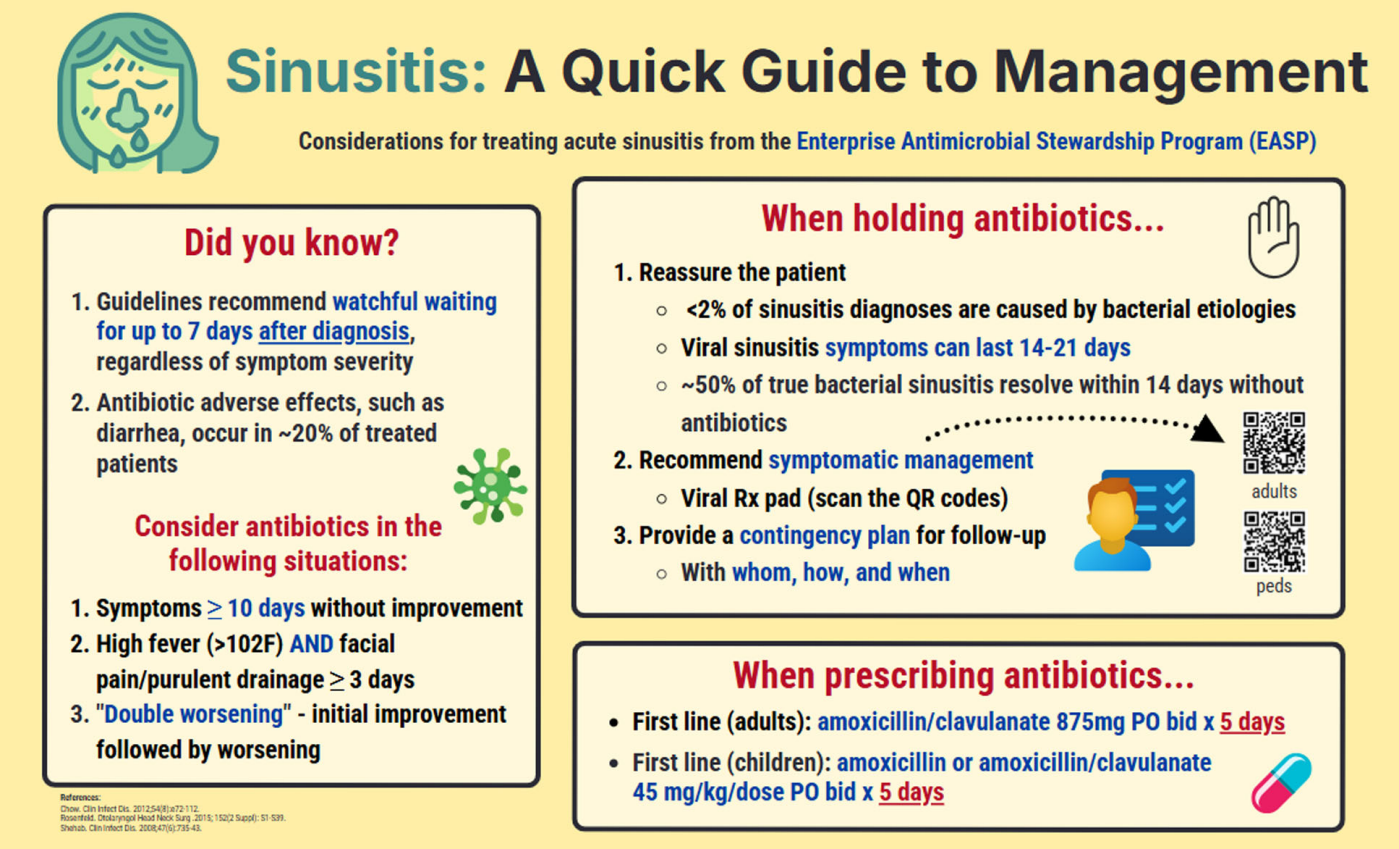# 68 Sporicidal Disinfection of High-Touch Surfaces to Reduce C. difficile Infection in Pediatric Oncology and Stem Cell Transplant Patients

**DOI:** 10.1017/ash.2026.10498

**Published:** 2026-06-23

**Authors:** Guyu Li, Laura Dinnes, Nipunie Rajapakse, Kourtney Gault

**Affiliations:** 1 Mayo Clinic

## Abstract

**Background:** Acute bacterial rhinosinusitis (ABRS) is a common, often self-limited condition that frequently leads to inappropriate antimicrobial prescribing in children. Continued reliance on the 2013 American Academy of Pediatrics (AAP) sinusitis guidelines and limited awareness of newer recommendations—particularly those supporting shorter treatment durations—have contributed to antibiotic overuse. As part of an enterprise-wide antimicrobial stewardship initiative, an intervention was implemented to support outpatient pediatric clinicians in identifying children most likely to benefit from antibiotics and in prescribing a 5-day course of guideline-concordant therapy consistent with the 2024-2027 AAP Red Book. We evaluated the impact of this intervention on prescribing practices and clinical outcomes. Methods A retrospective study was conducted among pediatric outpatients diagnosed with uncomplicated ABRS between January 1 and December 1, 2025 at an urban primary care pediatrics clinic within a large health system. The intervention consisted of a brief, targeted educational lecture delivered at a division meeting by pediatric antimicrobial stewardship leadership (pharmacist and physician), emphasizing updated guidance and promoting use of a sinusitis Epic order set. A one-page summary infographic was also distributed for reference. Data were extracted using Epic SlicerDicer and included patient demographics, clinical presentation, antibiotic selection and duration, use of the sinusitis order panel, and clinical outcomes. Outcomes before and after the intervention (May 14, 2025) were compared. Results A total of 99 pediatric patients with uncomplicated ABRS were included, with 55 treated before and 44 after the intervention. Use of the sinusitis order panel increased from 16% to 39% of encounters. The proportion of patients meeting diagnostic criteria for ABRS decreased slightly from 95% to 91%. Prescribing of 5-day antibiotic courses increased from 11% to 34%. There were no significant differences in prescribing provider type, antibiotic selection, or clinical outcomes—including Clostridioides difficile infection, return outpatient visits, or hospital admissions related to ABRS within 30 days—between patients receiving 5 versus <5 days of antibiotics (p<0.05). Conclusions A single, brief educational intervention paired with promotion of an Epic order set and summary infographic increased prescribing of shorter (5-day) antibiotic courses for pediatric ABRS without adversely affecting short-term clinical outcomes. Further studies are needed to assess the durability and long-term impact of this intervention on prescribing behavior.

**Figure:**